# Behavioral antecedents for handwashing in a low-income urban setting in Bangladesh: an exploratory study

**DOI:** 10.1186/s12889-017-4307-7

**Published:** 2017-05-05

**Authors:** Musarrat J. Rahman, Fosiul A. Nizame, Leanne Unicomb, Stephen P. Luby, Peter J. Winch

**Affiliations:** 1 International Center for Diarrheal Diseases Research, Bangladesh (icddr,b), Dhaka, Bangladesh; 2 Division of Infectious Diseases and Geographic Medicine, Stanford University, Stanford, CA USA; 3 Social and Behavioral Interventions (SBI) Program, Department of International Health, Johns Hopkins Bloomberg School of Public Health, Baltimore, MD USA

**Keywords:** Handwashing, Antecedent, Handwashing habit, Handwashing promotion, Health promotion, Behavior change

## Abstract

**Background:**

Health programs commonly promote handwashing by drawing attention to potential fecal contamination in the environment. The underlying assumption is that the thought of fecal contamination will result in disgust, and motivate people to wash their hands with soap. However, this has not proven sufficient to achieve high rates of handwashing with soap at key times. We argue that handwashing with soap is influenced by broader range of antecedents, many unrelated to fecal contamination, that indicate to people when and where to wash their hands. This exploratory study aimed to identify and characterize this broader range of handwashing antecedents for use in future handwashing promotion efforts.

**Methods:**

First, an initial list of behavioral antecedents was elicited through unstructured interviews, focus group discussions and observation with residents, from a low-income community in Dhaka, Bangladesh, who were also recipients of a handwashing intervention. Then, photographs representing three categories of behavioral antecedents were taken: activities of daily living, visual or tactile sensations, and handwashing-related hardware and activities. Finally, the research team conducted ranking exercises with a new set of participants, from the same area, to assess the perceived importance of each antecedent illustrated by the photographs. The research team probed about perceptions regarding how and why that particular antecedent, represented by the photograph, influences handwashing behavior.

**Results:**

After coming out of the bathroom and dirt (moyla) on hands were the two antecedents that ranked highest. In all the categories, intervention-related antecedents (three key times for handwashing which included handwashing after coming out of the bathroom, after cleaning a child’s anus and before food preparation; intervention provided items that included handwashing station, soapy water bottle, handwashing reminders from posters and community health provider visits) that were being promoted actively in this community were perceived favorably in the qualitative responses, but did not consistently rank higher than non-intervention items. However, many other antecedents were reported to influence when and where people wash their hands: cutting greasy fish, starting a meal, contact with oil and fat stuck to dishes, oil and lice from hair, sweat, unwashed vegetables, reminders from son and daughter or observing others wash hands, and observing the sunset.

**Conclusions:**

Beyond well-recognized antecedents related to fecal contact and dirt on hands, we identified a broader set of antecedents not reported in the literature. Adopting a handwashing promotional strategy to highlight existing antecedents that people themselves have identified as important can help inform the content of an intervention that is more relatable and effective in increasing handwashing practices.

## Background

Diarrhea-related diseases and pneumonia contribute significantly to mortality of children under five globally [1] and in Bangladesh [2]. Handwashing promotion can reduce the risk of both diarrhea [3] and respiratory infections [4, 5]. Specifically, handwashing can reduce diarrhea incidence by an approximate 30% - 48% ([3, 6]. Handwashing also can help control epidemics such as cholera and dysentery in urban slums [7] and refugee camps [8], reducing neonatal [9, 10] and maternal infections [11] and controlling pandemic influenza [12]. However, rates of handwashing are low in low-income countries [13]. While handwashing promotion interventions have demonstrated efficacy, handwashing behavior may not be sustained over time [14].

To be effective in achieving health outcomes, handwashing must be practiced at key times that include after stool contact, and before contact with food and water [13, 15]. There has been relatively little study of how specific intervention activities or other factors contribute to handwashing at key times [13, 15, 16], although there is considerable evidence globally that handwashing after fecal contact is not commonly practiced [13]. In a nationally-representative sample of Kenyan households, respondents were asked about their level of agreement with a number of statements on factors affecting handwashing. Agreement that handwashing is habitual was the variable most strongly associated with handwashing with soap, as measured by direct observation of handwashing in households included in the survey [17]. Arguably, handwashing with soap at key times and habitual handwashing more generally are influenced by features of the environment that act as signals, telling people when and where to wash their hands. Researchers refer to these features of the environment with a variety of terms: antecedent [18], trigger, cue [19–21] and stimulus [18].

In this paper, we adopt the term antecedent to refer to these features of the environment, and apply the framework of Applied Behavior Analysis- “the process of systematically applying interventions based upon principles of learning theory to improve socially significant behaviors to a meaningful degree, and to demonstrate that the interventions are responsible for improvement in behavior" [22]. Applied Behavioral Analysis has been used to identify and analyze antecedents and consequences of pro-environmental behaviors, and planning appropriate interventions [23]. The current study applies this methodology to study the relationship between handwashing behaviors and the environment. We contend that understanding antecedents of handwashing may open up new ways to promote handwashing at key times, by building upon or modifying existing antecedents.

For the purposes of this paper, we consider the term antecedent as synonymous with “antecedent environmental stimulus” [18] and we also consider the term handwashing to refer to any type of hand rinsing, with or without soap. Washing hands with water is common in the community from where the data for the current study was collected [24, 25]. While washing hands with soap has optimal health benefits, washing hands with water only is more beneficial than not washing hands at all [24]. We define a handwashing antecedent as a feature of the environment that 1) occurs prior to the person performing handwashing and 2) prompts a person to wash hands immediately (if the person is in a location where handwashing can be performed), or after developing the intention to wash hands.

In previous interventions that promoted handwashing by eliciting feelings of disgust, the underlying assumption was that seeing, smelling or thinking about fecal material induces disgust, thereby stimulating handwashing at key times [26–33]. Relatively little attention has been devoted to identifying antecedents of handwashing that are not related to feces or inducing feelings of disgust [34].

## Methods

### Aim

We sought to develop a comprehensive list of behavior antecedents for handwashing as a basis for future research on how to promote consistent handwashing. Our primary objective in this exploratory study was to elicit an initial list of behavioral antecedents from the community participants. Our secondary objective was to assess the perceived importance of each of these antecedents that were previously identified by community participants.

### Study setting

This study was conducted in the low-income Bauniabad neighborhood, Mirpur subdistrict in Dhaka City. The site was within one of three study arms (the vaccine and behavior change arm vaccine) in a randomized controlled trial named “Introduction of Cholera Vaccine in Bangladesh (ICVB)” [35, 36]. As part of this arm, study participants received handwashing stations (bright red water bucket with a tap), and soapy water bottles (1.5-l plastic bottles where participants could mix detergent and water to make soapy water for handwashing) [35, 36]. Community health promoters (CHPs) made regular visits to the households to monitor handwashing station usage and deliver promotion messages. Enrollees were also provided with posters depicting three key times for handwashing: after defecation, before food preparation and before eating.

### Study design

This study represents the initial stage of a sequential exploratory study design [37], and incorporated methods from ethnography and cognitive anthropology [38].

**Fig. 1:**
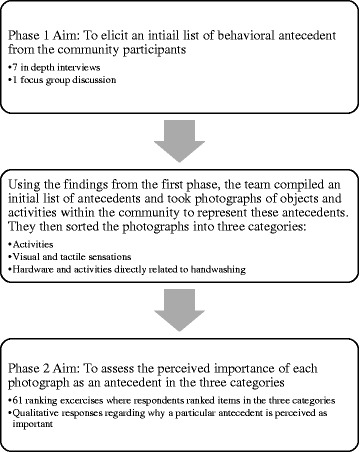
Flow Diagram of the study data collection phases

The study was conducted in two phases (Fig. 1). For the first phase, the team conducted 7 in-depth interviews and one focus group discussion to elicit an initial list of behavioral antecedents from the community participants.

Using the findings from the first phase, the team compiled an initial list of antecedents and took photographs of objects and activities within the community to represent these antecedents. They then sorted the photographs, or items from the exhaustive list into three categories:

1. Activities of daily living: coming from the bathroom, before a meal, fish cutting, cleaning a child’s anus after defecation, before food preparation, after cleaning dishes, sweeping, brushing hair, coming back from outside, before sleeping at night;
2. Visual or tactile sensations: dirt on hands, sweat, vegetables, handwashing station and soapy water bottle (intervention provided), evening sky, bedsheets, money; and
3. Hardware and activities directly related to handwashing: handwashing station, promotion visit from a CHP, watching someone else wash hands, intervention poster about handwashing, and reminders from a) son or daughter, b) a friend or neighbor, or c) an unknown person to wash hands.

Each of these three categories contained “intervention-related antecedents”. These included the three key times that were being promoted as part of the ongoing intervention (coming from the bathroom, cleaning a child’s anus after defecation, before food preparation), handwashing station, soapy water bottle, handwashing reminders from posters and community health provider visits.

For phase two, the team showed these photographs to 61 participants and conducted a separate ranking exercise for each category to assess the perceived importance of each photograph as an antecedent.

### Sampling and data collection

The data collection team included first author and research assistants. Five field research assistants in total aided with data collection. Two of the research assistants were trained professionals from icddr,b and three other research assistants were trained by the researcher. The FGD was moderated by one of the trained professionals from icddr,b and the notes were taken by one of the research assistants trained by the researcher.

The sampling in both Phase 1 and Phase 2 was purposive. We relied on the CHPs that were working in the neighborhood as part of the ICVB study to help us gain entrée to the households, to establish rapport with the respondents, and to locate respondents who were willing to participate. Study participants were all females residing in the urban slum who had received the intervention. Men were not available during the day at the time of data collection.

For preparation for the first phase, the data collection team first visited different parts of the study area for two weeks and took notes to identify features of the environment and objects and activities to help develop probes for the qualitative interviews. Once the interview guidelines were finalized, data collection for the first phase commenced.

As part of the all in-depth interviews and the focus group discussion, within the first phase, the data collection team asked about daily activities, and probed about activities and objects in participants’ daily lives that influence them to move toward handwashing facilities. The main aim was to identify and note the specific antecedent. For example, within a discussion of daily routine, if there was mention of coming back from outside and freshening up, the interviewer noted down “coming back from outside” as an antecedent.

Antecedents detected were photographed and used for the ranking exercises. For the second phase, photographs representing antecedents were shown to the participants during the separate ranking exercises for each category, to get a sense of what influenced handwashing behavior. For each of the exercises, we laid the photographs in front of the participant and asked them to identify which photograph represented an antecedent that most influenced them to wash hands and then asked them to order remaining photos all the way through to least influential from most influential. Additionally, for each ranking exercise, we also took detailed notes of their verbal reactions and probed about their perceptions regarding how and why that particular photograph influences handwashing behavior.

All interviews were conducted in Bengali. The qualitative responses were also noted in Bengali, and later translated to English.

Throughout this study, we asked the participants about what makes them develop the intention to wash hands with water and with or without soap. Even though soap is optimal for disease prevention, and it is known that the sight of soap can remind people to wash their hands, we wanted to elicit a full list of handwashing antecedents other than just soap. We did not probe on handwashing with soap in this initial exploratory study unless the participant mentioned it without prompting.

### Sample size

Since this was a small, pilot study conducted for the purposes of hypothesis generation, and we had limitations in personnel and budget, we did not attempt to make population level inferences. For phase one, we focused on generating, from the study participants, terms and definitions applicable in this social and cultural setting; we were not interested in assessing individual opinions or perspectives at this stage. Cultural Consensus Theory, as described by Romney et al. (1986) [39] provides a statistical justification for small sample sizes (4–10 respondents) when the researcher is examining forms of cultural knowledge with high inter-respondent agreement such as the names of the days of the week. We conducted the first round of interviews with 7 people, based in part on a limited time and personnel during the initial phase of the study.

We conducted one single focus group as a quick check, to determine if any of the terms or antecedents mentioned in the individual interviews were specific to individuals, or idiosyncratic.

In Phase 2, we were no longer interested in eliciting terms for antecedents, but rather in exploring the relative importance of the different antecedents from the perspective of the respondents, and the reasons for their ratings of importance. We anticipated that individual characteristics such as age, level of education, occupation and type of housing might affect the importance of different antecedents, but we did not design the study as a formal assessment of the influence of individual characteristics on ratings of importance. Cultural Consensus Theory suggests that at least 30 respondents are needed when the anticipated average cultural competence is below 0.5. We doubled this to give a sample size of 60 as we anticipated that there might be considerable variation. We ended up interviewing 61 persons.

### Data analysis

For the first phase of data collection, the team maintained a list of antecedents. After each interview, and then focus group discussion, the team members would add to the list based on the notes from that interview. The team would hold discussions among themselves to identify what would count as an antecedent, and because we kept our criteria very broad, we incorporated activities such as coming back from outside, or evening sky.

For the second phase of data collection, we collected quantitative and qualitative data.

The quantitative rankings are reported as mean ranks. We averaged the ranking for each photograph and calculated the standard deviations for each rank. We also calculated standard deviations as an indicator of agreement, or high consensus, among participants.

The ranking exercise data were interpreted through the lens of prototype theory. Prototype Theory (graded categorization) was first described in the 1970s [40]. As per this theory, if we ask people about when and where they wash hands, they will first mention more prototypical situations for handwashing, the conventional and accepted occasions for handwashing. People may not mention, or be aware of less prototypical situations where they wash hands. However, the less prototypical items may play important roles in making handwashing behavior habitual. It is possible that these less prototypical items ranked lower, but are still important for further exploration. This is why, we used additional criteria, in addition to high rank and low standard deviation, to identify antecedents that warrant further investigation. These criteria are explained in the Discussion section.

Our interpretations of the quantitative ranking data were informed by complementary qualitative data. We summarized the reasons for the ranking of each type of antecedent examined in the exercise. We then compiled the qualitative responses of all participants. For example, for sweat, we listed everything that all respondents mentioned about sweat and why they felt that sweat is an important antecedent. Then we looked for patterns of similarities and differences regarding why a participant perceived an antecedent to be effective.

## Results

Most participants were within the age range of 20–40 years and had no education or education till class 5 (Table 1). After coming out of the bathroom and dirt (*moyla*) on hands were the two antecedents that ranked highest in two of the ranking categories (activities of daily living identified as antecedents and visual and tactile sensations identified as antecedents). They also had lower standard deviations, indicating higher consensus about their ranking. Ranking was less consistent for the category for hardware and activities directly related to handwashing (Table 2).

**Table 1:** Demographic information for ranking exercise participants (*N* = 61)

Characteristics	Total (*N* = 61)
Participants’ age (years)	
Less than 20	5
20 to 29	22
30 to 39	18
40 to 49	12
50 and more	4
Participants’ education	
No education	28
Till Class 5	14
More than Class 5	19
Monthly Income in Bangladeshi taka (USD)	
1500 to 5000 (10 to 63)	16
5500 to 10,000 (69 to 125)	33
10,500 to 15,000 (131 to 188)	8
Above 15,500 (above 194)	4

**Table 2:** Mean Ranks for Antecedents

Rank	Activities identified as antecedents		Visual or Tactile sensations identified as antecedents		Hardware and activities directly related to handwashing	
	Description	Mean Rank (SD)	Description	Mean Rank (SD)	Description	Mean Rank (SD)
1	Coming out of bathroom	1.13 (0.74)	Dirt on Hands	1.31 (0.92)	Handwashing Station	2.72 (1.86)
2	Before Meal	3.07 (1.57)	Sweat	3.56 (1.61)	Community Health Providers	3.26 (1.91)
3	Fish Cutting	4.21 (1.44)	Vegetables	3.75 (1.77)	Reminder from Son or Daughter	3.48 (1.62)
4	Cleaning Child’s anus after defecation	4.79 (2.86)	Handwashing Station	3.93 (1.81)	Watching someone else wash hands	3.85 (1.67)
5	Before Food Preparation	4.87 (2.03)	Soapy Water Bottle	4.95 (1.54)	Reminder from Poster	3.97 (1.95)
6	After Cleaning Dishes	5.59 (1.94)	Evening Sky	5.23 (1.60)	Reminder from Friend or Neighbor	4.43 (1.54)
7	Sweeping	7.30 (1.98)	Bedsheet	6.16 (1.72)	Reminder from an unknown person	6.31 (1.31)
8	Brushing Hair	7.44 (1.67)	Money	7.13 (1.60)		
9	Coming Back From Outside	7.61 (1.43)				
10	Before Sleeping At Night	8.98 (1.40)				

Intervention items ranked higher than the non-intervention items; however the standard deviations were high across all items, possibly indicating lower consensus on the rankings. The qualitative responses provided context and explanations for why different participants seemed to value different antecedents.

### Activities of daily living identified as antecedents

Coming out of the bathroom was consistently ranked highly by participants, and was connected to comments on germs, smell and fear of sickness. One participant mentioned that it is natural and too obvious to warrant further explanation.

*“After coming out of bathroom, maybe I will eat something and germs will get into my stomach. So I wash to stop that”*

“*There are germs in the bathroom and the hands smell* “

*"This is most important, because right after waking up in the morning, first thing I do is go to bathroom and then I wash hands”*

Handwashing before a meal ranked second in the list. In Bangladesh the practice is to eat with hands so participants described the need to wash hands prior to eating as an obvious time, and that it was a habit or *obbhyash* to do so in order to remove dirt (*moyla*) and sicknesses. The word “*moyla*” in Bengali refers to dust and dirt, and more broadly to anything containing germs, or any kind of dirtiness. It can be translated as dirt, grime, soil or filth.

*“I always have to do this. It’s obvious"*

*"There might be dirt (*moyla) *in the nails, so we will wash hands till hands are clean and then we will sit down to eat*"

"*The food won’t be contaminated and sicknesses won’t enter my stomach"*

When it came to cutting raw fish before cooking, respondents cited the accompanying greasy sensation and smell that remained on hands compelled them to wash their hands.

“*Fish can make hands smell and then there is a disgusting smell when I go to eat something. The smell is stuck to my hands”*

Cleaning a child’s anus after defecation was the fourth most common activity antecedent, connected by study participants to factors affecting illness occurrence and prevention, such as the desire to maintain children’s health and reduce exposure to germs and illness.

When we showed pictures of a woman in the kitchen preparing food, and asked whether they would wash hands before food preparation, participants mentioned that they might wash hands after food preparation, and not necessarily before. Specifically, they mentioned that handling vegetables, fish and spices during food preparation will compel them to wash hands.

The bottom five ranking antecedents included washing dishes, sweeping, brushing hair, coming back from outside, and before sleeping at night. Among these, washing dishes and brushing hair elicited inconsistent responses regarding their importance as triggers to handwashing. While some participants said that the hands get washed in the process of cleaning dishes, others highlighted that the process of cleaning dishes can result in oil or fat from the leftover food and the *kali* or black burnt-on or scorch stains from the bottom of pots transferring to hands. Similarly, for hair brushing, some participants said that it is not necessary to wash hands after brushing hair, but some connected this activity with coming into contact with lice and oil which led them to wash hands. Bangladeshi women commonly apply coconut oil to their hair, and the odor of coconut oil is distinct.

*“I feel disgusted after touching hair. Because we kill lice after putting oil in hair that is why I wash my hands”*

*“If I brush hair, oil will get on hands from the hair oil in our hair. So I wash hands”*

Sweeping and coming back from outside were themes that were connected with evening time, dust, dirt and the need to freshen up. Some said that the sight of the tap and soap after coming back from outside will trigger the need to wash hands.

*“In the evening I will also sweep and if there is too much* moyla *then I will wash”*

Most participants said that they do not routinely wash hands before going to bed at night, but those who did, mentioned that it is a habit and that they like freshening up before going to sleep.

### Visual or tactile sensations identified as antecedents

Dirt (*moyla*) on hands was described by all study participants as a very strong antecedent for handwashing.

“*Everyone will wash hands when the hands are dirty and have* moyla*. I feel disgusted if there is* moyla *on hands. Also we wash hands to avoid diarrhea”*

“*hands feel disgusting when* moyla *gets there*”

Wiping sweat from forehead came up as a strong antecedent for handwashing. Participants explained that sweat prompted them to wash their hands because sweat results in itching, it is sticky and salty and it gives off a bad smell. Participants claimed they felt the need to clean up after wiping sweat.

We showed a picture of a woman holding vegetables in the market. Some participants interpreted this picture as “after cutting vegetables” as opposed to touching vegetables. Some mentioned that they will wash hands after cutting vegetables.

Participants spoke favorably of the intervention hardware, the handwashing station and soapy water bottle, that acted as visual antecedents. They found the red colored handwashing station attractive to look at and the placement of handwashing stations increased convenience. The soapy water bottle was kept as part of the handwashing station and so it also ranked highly. Participants mentioned that the soapy water was convenient because it was mixed and ready for use.

Participants connected evening with time for prayer, finishing household work and asking children to freshen up and start studying. Participants also explained how evening is the time when they come back from outside and there is a need to freshen up, because one was exposed to dust and dirt from the streets outside.

*“The streets have dust and dirt that get into the body”*

*“As soon as the call to prayer happens, I will perform ablution with water and then pray. I always do this”*

*" In the evening I will freshen up and freshen my children and wash their hands and feet and send them to study"*

*"This is also important. At evening I finish my cooking, finish working, I have to wash my hands and feet then "*

Items such as pictures of hands touching bedsheets or money ranked consistently low overall, and most participants said that it is not necessary to wash hands after touching bedsheets or money, even though they were mentioned in the first round of interviews.

### Hardware and activities directly related to handwashing

This exercise involved ranking candidate reminders for people to wash hands from posters, home visits by CHPs and other reminders.

In the qualitative responses, participants continued to speak favorably regarding the intervention hardware and promotional activities. Most participants mentioned that the handwashing station acts as a strong reminder for washing hands in addition to reminders from children and CHPs.

“*Since we come from an uneducated environment, the CHPs are teaching us and that encourages us”*

*“The neighbors will not be around always, they will come and go, but my daughter will always be inside the house and so she reminds us the most”*

*" Children study so they know "*

Participants spoke favorably of the posters, however some mentioned that they did not like hanging the posters in their homes. During our observations, we also found that many had put away the posters inside drawers. One participant mentioned a religious injunction against hanging such posters near where one performs ablutions and prays.

“*For Islamic prayer ritual, I turn it around, but there are some next to the (handwashing) station, so those remind me to wash hands*”

Watching someone else wash hands, reminders from friends and neighbors, and unknown persons elicited mixed responses. Some mentioned that this can be a good motivator, but most explained how they are the ones reminding others and some became defensive and mentioned that they do not need others reminding them.

“*Yes I tell my neighbors. I tell them that the handwashing station has been given to you just to keep for show? You should wash hands. You can learn well because of this”*

*“I hear in it one ear and take it out from the other ear “meaning “I don’t pay attention or importance to them”*

*"Their business is their business and mine is mine. I don’t pay attention to what they are doing"*

## Discussion

The current study identified two existing antecedents that were unanimously perceived as prototypical for handwashing— coming out of the bathroom and dirt or *moyla* on hands. In addition, the current study identified a host of other antecedents that ranked lower and were less prototypical but may still represent antecedents that can be useful for future handwashing promotion.

Prototype theory suggests that people may assign a high ranking to an item in a category because it has prototypical characteristics, not necessarily because it is important or influential [40]. Based on this premise, we took into account several other criteria beyond the results of the ranking exercise to define a set of antecedents that warrant further exploration in future studies.

We were expecting intervention-related antecedents to rank relatively higher than non-intervention antecedents (since the intervention antecedents were being promoted actively in this community) but this was not strictly the case. Even though participants spoke favorably of the intervention antecedents in qualitative responses, perhaps a result of courtesy or novelty biases, intervention items did not consistently rank higher than non-intervention items in the ranking exercises. This is why we first considered antecedents that ranked higher than the lowest ranking intervention antecedent that were actively promoted in the intervention activities. Items that ranked higher than the lowest intervention items may warrant further consideration since they were likely competing with antecedents that were being promoted actively in this community. For example, in the case of the first category, the intervention-related antecedent that ranked the lowest was food preparation which ranked at 5. Before meal and fish cutting were the two antecedents that were not related to the intervention that ranked higher than the food preparation, so we took these into consideration.

We secondly considered antecedents for which the qualitative responses highlighted certain nuances in interpretation that must have affected the ranking decisions deeming the corresponding rank as inconclusive. We thirdly considered antecedents that came up as relevant repeatedly in connection to qualitative responses to other antecedents.

### Potential handwashing antecedents for future handwashing promotion

As per our first criterion for identifying potentially important antecedents, cutting fish, before meals, dirt on hands, sweat and vegetables, reminder from son or daughter, and watching someone else wash hands are worth considering in future handwashing promotional activities. Cutting fish, dirt on hands, sweat and vegetables are antecedents that were connected by respondents with the sensory feeling of disgust. The relationship between disgust and handwashing has been identified to be both strong and universal [26, 41] and possibly having an evolutionary basis [27]. For handwashing promotion, disgust has conventionally been linked with feces. However, participants in the current study rarely mentioned feces as a handwashing antecedent, despite its prominence in the handwashing promotion literature. Washing hands before a meal (an antecedent connected with the habit and cultural practice of eating with hands), reminder from son and daughter and watching someone else wash hands are non-disgust antecedents that can also be considered for further promotion.

As per our second criterion, for some of the antecedents, the qualitative responses highlighted how nuances in interpretation of the photo images might have affected the rankings. For example, participants mentioned that simply brushing hair by itself does not compel one to wash hands, but many connected brushing hair with hair oil and lice on hands which were cited as good reasons for washing hands. Cleaning dishes, similarly, was equated with leaving oil, fat and scorch marks or *kali* from the pans on hands. We know that disgust is an important motivator for handwashing [28] and therefore, oil, fat and *kali* stuck to dishes, and oil and lice from hair seem to be important disgust-related antecedents for washing hands (even though brushing hair and after cleaning dishes ranked low) that warrant further exploration.

As per our third criterion, one particular antecedent, evening sky or observing the sunset, was frequently linked with other antecedents (and when asked about other antecedents, they were frequently linked with evening sky). Evening time is marked by the call to prayer, and was linked with coming back from outside where there is exposure to dust and dirt while walking or working. Evening sky and coming back from outside were both linked to the sight of the tap on entering the house. Evening further marks the time of day to finish household chores such as cooking, sweeping the house, and asking children to freshen up, all of which contribute to adults’ need to freshen up. It seems that during the evening, a confluence of different antecedents may work together to increase the likelihood of washing hands, so framing handwashing promotion messages around evening time may be more effective than solely focusing on high risk times [24].

### Study limitations

The current study has associated limitations. First, psychologists emphasize that a large proportion of our triggers are unconscious. Handwashing interventions try to inculcate the behavior to the extent that it becomes a habit practiced with a high degree of automaticity [42]. We were able to obtain information only on antecedents that participants easily recalled from their own experiences; habitual behavior is less often easily recalled. To obtain antecedents that may not be so obvious or that respondents might not be conscious of, the study team probed participants extensively in the first round of interviews to obtain a list that was as exhaustive as possible. The first round of interviews stopped only when data saturation was reached and participants were no longer mentioning new antecedents.

A second limitation is that researchers have noted that participants may choose to respond to questions in an interview based on what they perceive the interviewer wants to hear, a phenomenon often referred to as courtesy bias [43, 44]. In this study, study participants may have perceived that the study team wanted to hear that they washed hands during the key times promoted by the intervention and because of the hardware provided by the intervention. It is possible that they assumed the study team was less interested in other times or places that preceded handwashing. This is a difficult bias to adequately address. However, using a ranking method, we obtained some antecedents that were not intervention related that were identified as important by the community, and these antecedents might be important for future consideration.

A third limitation is that the behavioral antecedents identified in this community may be quite different to behavioral antecedents in other communities. To address this concern, future studies can similarly elicit such lists which will differ from one context to another. A fourth limitation is that the current study provides data only on women, primarily because, handwashing interventions are aimed at caregivers. Young children and infants have developing immune systems and attacks to their immune systems can lead to irreversible growth faltering, which is why caregiver’s hand hygiene is particularly important to prevent growth faltering in children. However, future studies should elicit similar lists for men, so that handwashing interventions can include them also.

A final limitation is that the current study did not focus on washing hands with soap, but instead, focused on what compels someone to form the intention to wash hands, even if using water only. Antecedents to rinsing hands with water, with or without soap, are relevant antecedents for the general practice of handwashing which with behavioral coaching or support could include soap.

Despite these concerns, this study makes a valuable contribution to the literature, because it has identified a larger set of antecedents to be investigated and tested in future research on how to most effectively promote regular handwashing with soap.

### Strategies for promoting handwashing

These newly recognized antecedents can be used in strategies for handwashing promotion. Our study found that people hide posters out of religious concerns. Some consider pictures of human figures as unreligious. In such cases, intervention materials might replace human figures with pictures of more relatable antecedents identified as part of the current research to elicit disgust (oil and fat and *kali* from dishes, oil and lice in hair, smell of fish, *moyla* on hands, sweat and cutting vegetables). Posters could be fixed at the entrance to the kitchen and say “you are now entering food preparation area…” with pictures of relatable antecedents to remind people that entering the kitchen means that they need to wash hands.

A further strategy can be to draw analogies [45, 46]. Promoters can link “community key times” to “study key times.” For example, CHPs can say “Of course, everyone washes their hands after touching fish, after getting oil and lice on their hands, but other important times to wash hands are before food preparation….”. Here, touching fish and getting lice or oil on hands are the naturally occurring antecedents that we seek to link to the key times for handwashing such as before food preparation [24]. Furthermore, evening marks a temporal threshold that can be utilized as a reminder in an intervention. For example, posters can read “Just as you wash your hands after coming home in the evening, so too you need to wash your hands in the morning and in the afternoon before preparing meals” and "the call to prayer in the evening is a good reminder to freshen up, similarly, the time before preparing food in the mornings or afternoons is a good reminder to wash hands.".

## Conclusions

Efforts to encourage handwashing at key times which have been promoted for the last two decades have not resulted in high levels of handwashing with soap [25, 47, 48]. Alternatively, adopting a strategy to highlight existing antecedents that people themselves have identified as important can help devise an intervention that is more relatable and effective. Future programs can use these new antecedents to design, pilot and test behavior change messages and promotional strategies to increase handwashing as a way to have hands cleaned more frequently during the day. Even if handwashing does not occur at key times, an overall increase in handwashing frequency may improve health.
